# A Bizarre Cause of Acute Appendicitis in a Pediatric Patient: An Ingested Tooth

**DOI:** 10.3390/children10010108

**Published:** 2023-01-04

**Authors:** Zenon Pogorelić, Tin Čohadžić

**Affiliations:** 1 Department of Pediatric Surgery, University Hospital of Split, Spinčićeva 1, 21000 Split, Croatia; 2 Department of Surgery, School of Medicine, University of Split, Šoltanska 2, 21000 Split, Croatia

**Keywords:** acute appendicitis, children, teeth, foreign body

## Abstract

(1) Background: Among all possible causes, foreign bodies are the rarest cause of appendicitis in the pediatric population. In the majority of cases, ingested foreign bodies pass through the gastrointestinal tract without causing symptoms. However, those foreign bodies that pass through the lumen of the vermiform appendix cannot re-enter the colon and may cause acute appendicitis. So far, various foreign bodies have been described to enter the appendix and cause acute appendicitis, such as seeds, needles, toothpicks, plant material, or even hair. Tooth or dental implants as a cause of acute appendicitis have been described in only a few cases. To our knowledge, this is the first described case of the tooth causing acute appendicitis in the pediatric population. (2) Case presentation: A 14-year-old male patient presented to the emergency department complaining of pain in the right lower quadrant of the abdomen and vomiting that persisted for approximately 24 h. Until then, the patient was healthy and had no concomitant diseases. Physical examination revealed guarding and tenderness in the right lower abdominal quadrant. The white blood cell count was 17.1 × 10^9^/L with a neutrophil count of 91.1% and a C-reactive protein of 39.3 mg/dL. Ultrasonography of the abdomen revealed a thickened, inflamed appendix with a diameter of 11 mm and free periappendicular fluid. A 9 × 6 mm foreign body at the base of the appendix was visualized. The patient was diagnosed with acute appendicitis, and an emergency laparoscopic appendectomy was performed on the same day. Intraoperatively, gangrenous appendicitis was noted and removed without complications. Upon examination of the removed specimen, it was determined that the patient’s appendicitis had been caused by an ingested tooth. As it later turned out, the patient had broken a lateral incisor while playing sports the day before admission without knowing that he had swallowed it. The patient recovered well and was discharged the day after surgery. (3) Conclusion: Although an extremely rare event, acute appendicitis can be caused by a swallowed tooth. This case highlights the importance of a thorough history in pediatric patients who present to the emergency department with suspected acute appendicitis to determine if there is a precipitating event that may have caused acute appendicitis.

## 1. Introduction

Acute appendicitis is one of the most common causes of abdominal emergency surgery in the pediatric population. Acute appendicitis can occur at any age, but the peak incidence in children is between 11 and 12 years of age [1]. It is very rare in children under five years of age, especially in children under two years of age. The majority of children, nearly 80%, in this age group have perforated appendicitis and diffuse peritonitis at the time of presentation to the emergency department [2,3].

The majority of cases of acute appendicitis can be diagnosed by physical examination. In addition to physical examination and image diagnosis, numerous laboratory markers such as white blood cells, neutrophil granulocytes, C-reactive protein, interleukins, sodium, bilirubin, red blood cell distribution width, mean platelet volume, etc. [4,5,6,7,8], as well as various scoring scales, help us to diagnose acute appendicitis [9,10,11]. Although acute appendicitis is the most common surgical abdominal pathology in children, it is still a challenge and diagnostic dilemma for pediatric surgeons despite all available diagnostic modalities.

Although there are numerous published reports on the conservative management of acute appendicitis, appendectomy is still the gold standard of treatment in most centers [12,13,14,15]. Recently, laparoscopic appendectomy has almost completely replaced open appendectomy [14,16]. After a laparoscopic appendectomy, patients recover very quickly in the majority of cases and can be discharged within 24 h after the procedure [16]. The number of complications, readmissions, and reoperations after laparoscopic appendectomy is low [16].

The exact cause of acute appendicitis remains unknown in approximately 60% of cases [17,18,19,20,21]. In the remaining cases, it is caused by lumen obstruction. Obstruction of the lumen is most commonly caused by fecal matter or lymphoid hyperplasia; rarely, appendicitis is caused by tumors, intestinal parasites, or foreign bodies [18,19,20]. In the majority of cases, ingested foreign bodies pass through the gastrointestinal tract without causing symptoms. However, foreign bodies that pass through the lumen of the vermiform appendix cannot re-enter the colon and may cause acute appendicitis [19,21]. The prevalence of acute appendicitis caused by foreign bodies is 0.0005% [21]. To date, numerous foreign bodies, such as small metal objects, animal hair, toothpicks, bullets, piercings, needles, stones, seeds, or pins, have been described as the cause of acute appendicitis [21,22,23,24,25,26,27]. Very few cases of acute appendicitis have been caused by dental objects, most commonly dental implants, dental vires, or dental crowns. Appendicitis caused by an ingested tooth is extremely rare, and not a single case has been reported in the pediatric population [28,29].

Here, we present a case of a 14-year-old male pediatric patient who was admitted to the emergency department with acute appendicitis due to an accidentally swallowed tooth.

## 2. Case Report

A 14-year-old male patient presented to the emergency department complaining of right lower quadrant abdominal pain and vomiting that had persisted for approximately 24 h. Initially, the pain was localized periumbilically, whereas several hours later, the pain shifted to the right lower quadrant of the abdomen. The patient complained of nausea, vomited stomach contents several times, and had no appetite. Until then, the patient was healthy and had no concomitant diseases.

**Figure 1:**
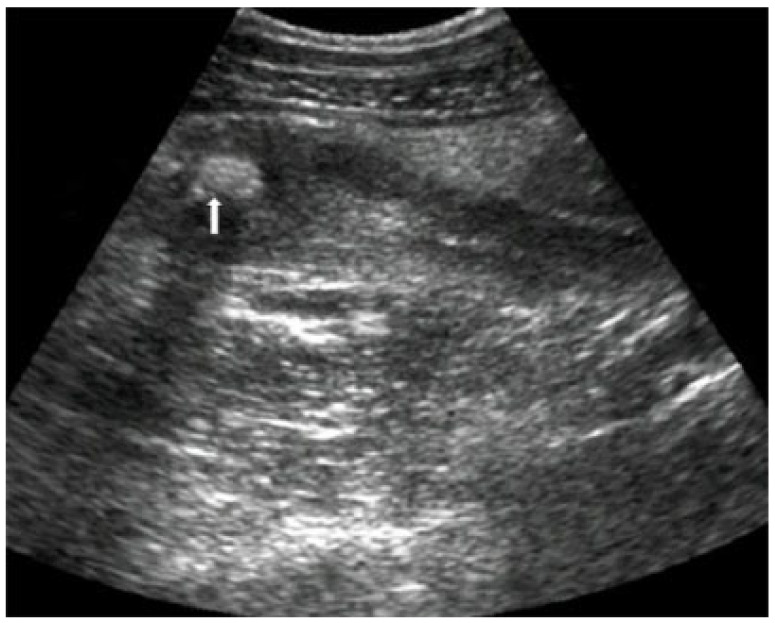
Abdominal ultrasound—Acute appendicitis with visible foreign body (arrow).

Physical examination revealed guarding and tenderness in the right quadrant of the lower abdomen. Blumberg, Grassman, and Rovsing’s signs were positive. The patient’s vital signs were within normal limits, while the body temperature measured axillary was 37.7 °C. Laboratory analysis of the blood sample revealed elevated levels of acute inflammatory reactants. The white blood cell count was 17.1 × 10^9^/L with a neutrophil count of 91.1% and a C-reactive protein of 39.3 mg/dL. Ultrasonography of the abdomen revealed a non-compressible, thickened, inflamed appendix 11 mm in diameter with free periappendicular fluid. A foreign body measuring 9 × 6 mm at the base of the appendix was visualized (Figure 1).

**Figure 2:**
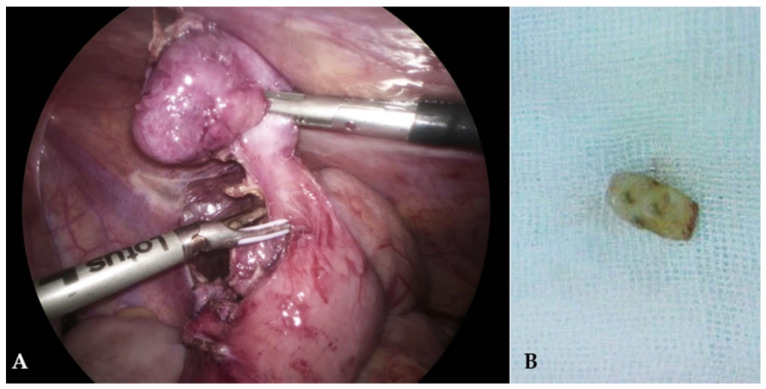
Intraoperative findings: (**A**)—Gangrenous acute appendicitis; (**B**)—Removed tooth from the removed appendiceal specimen.

The Appendicitis Inflammatory Response (AIR) score was 10, placing the patient in the high-risk group for acute appendicitis. Finally, the patient was diagnosed with acute appendicitis, and a three-approach laparoscopic appendectomy was performed the same day. Intraoperatively, gangrenous appendicitis (Figure 2A) was noted and removed without complications. During the dissection of the appendix, the surgeon noted a hard formation at the base of the appendix. Upon examination of the removed specimen, it was determined that the patient’s appendicitis had been caused by an ingested tooth (Figure 2B). The final pathohistological diagnosis was gangrenous appendicitis. As it later turned out, the patient had broken a lateral incisor while playing sports the day before admission without knowing that he had swallowed it.

The patient was admitted to the Department of Pediatric Surgery. Postoperatively, infusions of crystalloid solutions and analgesics were prescribed. Oral fluids were started a few hours after surgery, followed by a light diet, which the patient tolerated well. Throughout the hospital stay, the patient was in good general condition and had no fever. The patient was discharged 16 h after surgery in good general condition. The patient recovered quickly, and at the 7-day follow-up in the outpatient clinic, the stitches were removed.

## 3. Discussion

This case report presents a rare and bizarre case of acute appendicitis caused by an ingested part of the tooth in a 14-year-old pediatric patient. On the day of the patient’s examination, the missing lateral incisor went unnoticed, and anamnesis after surgery revealed that the patient had broken a lateral incisor while playing sports the day before admission to the hospital. This case confirms that the rare cause of acute appendicitis may be ingestion of a foreign body, which can, in some cases, be complicated by peritonitis, perforation or even abscess.

It has previously been reported that various foreign bodies have been found to cause perforation in 75% of the cases, including fish bones, animal bones, or bone fragments and toothpicks [22]. However, a whole tooth or part of it in the appendix is a very rare cause of acute appendicitis. Generally, the prevalence of appendicitis due to foreign bodies is very low. The results obtained by Collins et al. reported that out of 71,000 appendectomies, 51.8% were caused by obstruction, of which most of them were parasitic worms or faecoliths and only 5.5% of them were considered unusual foreign bodies [11,29]. The ingestion of foreign bodies generally does not cause gastrointestinal complications, such as acute appendicitis, and it can pass within a week with less than a 1% rate of complications [27]. Symptom onset and the entry of a foreign body into the appendix are often dependent on the size and shape of an object and the anatomical position of the appendix [21]. Most of the foreign bodies are sharp and elongated and are more likely to cause perforation of the appendix and peritonitis. In the case of blunt foreign bodies, the obstruction of the appendiceal lumen appears, which remains dormant for a period of time. After the obstruction, the peristaltic motion needed to move it back into the caecum is absent, and the chances for appendicitis are high [21,23,29].

The most common cause of appendiceal obstruction is fecalith, followed by intestinal parasites and rarely tumors. Foreign bodies as a possible cause of acute appendicitis have been described in several recent studies (most commonly part of bone or toothpick). Other less common foreign bodies such as earrings, needles, and dental objects have been described [23,24,27,30,31,32]. A recent study describes how a detailed history revealed that the patient had swallowed a dental crown. After the dental crown showed up on the CT scan, it was successfully removed by a surgical method [27].

Conservative management of uncomplicated appendicitis with antibiotics has been associated with reduced complication rates and hospitalization [13,15]. However, previous studies showed that there are several risk factors associated with a higher recurrence rate of appendicitis for those who initially undergo non-operative management. Some of the most valuable factors pointing at the necessity of operative management include an appendix diameter greater than 1 cm and the obstruction of the appendicular lumen by a fecalith [23,27]. The European Association for Endoscopic Surgery (EAES) guidelines command that cases of both uncomplicated and complicated acute appendicitis should follow laparoscopic appendectomy as a gold standard [16,23]. The open appendectomy approach can be used as well, but, as known, it increases the post-operative hospital stay and is missing the diagnostic perspective, which increases the incidence of negative appendectomies. Therefore, it is likely that the surgical approach is based on the clinical judgment and skill set of the operating surgeon [1,23]. Despite recently published data showing that antibiotics can be used for uncomplicated appendicitis, cases presenting with an obstruction or ingested foreign bodies are not amenable to non-operative treatment and should always be scheduled for laparoscopic appendectomy [33,34,35,36]. Abdominal ultrasound or computed tomography are very useful in such cases, and a thorough history and physical examination are very important to exclude the possibility of a foreign body as the cause of appendicitis [37,38].

## 4. Conclusions

Although it is an extremely rare event, acute appendicitis can be caused by an ingested tooth. This case highlights the importance of a thorough history in pediatric patients who present to the emergency department with suspected acute appendicitis to determine if there is a precipitating event that may have caused acute appendicitis, which is very important for treatment strategies.

## Data Availability

Not applicable.
